# Escalating Threat of Drug-Resistant Human Scabies: Current Insights and Future Directions

**DOI:** 10.3390/jcm13185511

**Published:** 2024-09-18

**Authors:** Thierry Simonart, Xuân-Lan Lam Hoai

**Affiliations:** 1 Department of Dermatology, Delta Hospital, CHIREC, Université Libre de Bruxelles, 1160 Bruxelles, Belgium; 2 Department of Dermatology, St Pierre—Brugmann—HUDERF University Hospitals, Université Libre de Bruxelles, 1000 Brussels, Belgium; xlamhoai@gmail.com

**Keywords:** scabies, resistance, permethrin, ivermectin, benzyl benzoate

## Abstract

**Background**: Scabies is a prevalent dermatological condition with significant public health implications. The recent rise in drug-resistant scabies presents new challenges for effective disease management and control. **Methods**: A comprehensive literature review was conducted using PubMed, Cochrane Library, and Web of Science. Studies published from 2000 to August 2024 were considered, focusing on those reporting drug-resistant scabies and advancements in treatment approaches. **Results**: Clinical studies, in vitro investigations, and case reports show significant resistance of human scabies to permethrin. Main resistance mechanisms involve genetic mutations in the mites’ voltage-gated sodium channels (VGSCs) and enhanced activity or expression of the detoxifying enzyme glutathione S-transferase (GST). Resistance to ivermectin and benzyle benzoate, although suggested by some authors, seems less obvious. The clinical evidence of widespread ivermectin resistance in human scabies infestations is lacking, despite indications of increased tolerance in laboratory settings and anecdotal reports of resistance in patients with crusted scabies. Benzyl benzoate resistance in scabies mites remains unconfirmed. **Conclusions**: Permethrin-resistant scabies is an escalating threat requiring new management strategies and updated guidelines. Infection control measures, alternative treatments, and ongoing research into new therapeutics are crucial to mitigate the impact of drug-resistant scabies.

## 1. Introduction

Scabies is a highly contagious skin infestation caused by the mite *Sarcoptes scabiei*. It is characterized by intense pruritus, particularly nocturnal, and excoriated papules or papulo-vesicles that are often symmetrically distributed [1,2,3]. Common sites of involvement include the axillae, interdigital spaces of the hands and feet, waistline, and buttocks. Physical examination may reveal characteristic burrow lines, formed as female scabies mites tunnel into the skin to deposit eggs [1,3].

While scabies is endemic globally, its distribution is heterogeneous [4]. Primarily transmitted through skin-to-skin contact, scabies outbreaks commonly occur in congregate settings such as prisons, shelters, nursing homes, and hospitals and other crowded areas [1,3]. High-income countries predominantly experience sporadic cases, with outbreaks primarily affecting institutionalized populations such as hospitals and residential care facilities for older people [1,3,4]. In contrast, low- and middle-income countries bear a heavier burden of disease due to factors such as limited access to effective treatment and increased promiscuity [4,5].

With an estimated 400 million cases worldwide [5], scabies constitutes a significant public health challenge. Beyond the immediate skin manifestations, secondary complications, including impetigo, necrotizing soft-tissue infections, rheumatic heart disease, rheumatic fever, and glomerulonephritis, may occur [1,3,5]. Moreover, the condition is associated with impaired quality of life and psychosocial distress [6], underscoring its multifaceted impact.

Several epidemiologic studies, along with local reports of scabies outbreaks, analysis of big data, and increases in sales of scabies treatments suggest that the incidence of scabies is increasing in several countries [4,7,8,9,10,11,12]. A recent study in the Netherlands reported a more than three-fold increase in scabies incidence from 2011 to 2020 [13]. In Spain, prescriptions for 5% permethrin cream for scabies increased six-fold between 2008 and 2021 [14]. This increase is thought to be related to increasing population density in urban settings, limited access to healthcare, migration, travel, and an aging population [12,15].

The traditional pillars of scabies treatment involve the use of topical permethrin 5% and oral ivermectin [1,3]. This strategy is still supported by expert recommendations as well as by local and international guidelines [1,3,16,17,18,19]. Topical treatments are cumbersome as they necessitate meticulous application to the entire skin surface for at least 8 h [18]. Permethrin cream requires reapplication after 7–10 days, frequently leading to suboptimal treatment adherence. This can result in persistent infection and increased transmission, particularly in crowded environments. However, several data suggest that the emergence of drug-resistant scabies has compromised the efficacy of some anti-parasitic drugs, posing a significant challenge to disease management [20,21,22]. This challenge to scabies management is further complicated by a heterogeneous approach to disease management among dermatologists [23]. Authors from the UK performed an analysis of different guidelines and revealed substantial variability in recommendations, particularly regarding prophylactic measures, anti-scabies treatment, infection control, and mass treatment strategies [24]. Treatment options also differ between elderly and pediatric patients with scabies. In children, permethrin is considered a first-line therapy against scabies, while oral ivermectin is generally used in mass drug administration programs in endemic communities or when topical treatments have failed or are contraindicated due to skin sensitivity or poor compliance [25,26,27].

This review aims to provide an updated overview of drug-resistant scabies, examining the mechanisms of resistance. Additionally, it discusses the implications for clinical practice and describes the current and future strategies for managing drug-resistant scabies.

## 2. Materials and Methods

A comprehensive literature search was conducted using PubMed, Cochrane Library, and Web of Science. Keywords included “scabies”, “resistance”, “resistant”, “permethrin”, “ivermectin”, “benzyl benzoate”, “treatment”, “guidelines”, “recommendations”, and “failure”. Articles in English or for which an English translation was available published between 2000 and August 2024 were reviewed. Inclusion criteria encompassed clinical and laboratory studies that reported on drug resistance in scabies, described mechanisms of resistance, or evaluated treatment efficacy in the context of resistance. A narrative synthesis approach was employed to summarize findings across studies.

## 3. Results

### 3.1. Incidence of Drug Resistance

Early clinical reports of scabies resistance to permethrin emerged in the early 2000s, highlighting significant concerns about the efficacy of this frontline treatment. Walton et al. conducted a survey in Australian Aboriginal communities, revealing numerous treatment failures with permethrin, which suggested emerging resistance [28]. Similarly, Currie et al. documented cases in Northern Australia where patients required multiple rounds of treatment or alternative therapies due to inadequate responses to permethrin, further emphasizing the clinical implications of resistance [29]. Subsequently, Pasay et al. corroborated these findings through a case report identifying specific genetic mutations in the mites’ voltage-gated sodium channels (VGSCs), which were linked to permethrin resistance [30]. In Europe, Meyersburg et al. described reduced susceptibility of scabies mites to permethrin in Austria, based on both clinical observations and laboratory evidence, and called for the evaluation of alternative treatment options [31]. In 2021, an Italian group observed that nearly two-thirds of patients who did not respond to permethrin showed improvement with another topical treatment, suggesting specific resistance to permethrin rather than issues of compliance [32]. In a 2024 study from local UK sexual health clinics, the authors showed a significant increase in the median number of treatments per scabies case per year over the past 7 years [33]. Case series and case reports from other authors from other geographic areas corroborate these findings [34,35,36,37,38,39]. Further supporting those findings, a meta-analysis evidenced a trend of increasing overall permethrin treatment failure in studies published in 2011 or later [22]. As a whole, the distribution of resistance appears to correlate with the intensity of scabies control programs and the widespread use of specific treatments [22,40]. Additionally, scabies patients with limited mobility and topical steroid use before diagnosis are at high risk of treatment resistance [41].

In vitro investigations have shown varying degrees of susceptibility of scabies mites to permethrin, depending on geographic and population differences. Acaricide sensitivity assays demonstrated significant reductions in permethrin susceptibility in mites collected from patients with treatment failures [42]. However, these in vitro data are challenged by another study showing that permethrin retains significant killing activity against scabies mites [43], emphasizing that resistance may not be uniformly widespread. These in vitro studies are critical as they provide a controlled environment to directly assess the sensitivity of scabies mites to permethrin, complementing clinical observations and highlighting the complexity of resistance patterns.

Although there is some evidence that the in vitro tolerance of scabies mites to ivermectin in scabies-endemic communities is increasing [42], the clinical reports of ivermectin resistance among patients with scabies remain largely anecdotal, have been reported in patients with crusted scabies who received repeated and prolonged treatment, and are not necessarily recent [29,44]. Moreover, recent studies continue to demonstrate high levels of mite clearance and sustained efficacy after ivermectin therapy [45,46].

Despite a long-standing use of benzyl benzoate, one of the older treatments for scabies, resistance to benzyl benzoate seems limited. An anecdotal study, lacking robust methodology, has reported treatment failure with benzyl benzoate, characterized by persistent pruritus and infestation despite adherence to recommended regimens [47].

### 3.2. Mechanisms of Drug Resistance

The different mechanisms underlying drug resistance in *Sarcoptes scabiei* are complex and multifactorial and may involve the mite’s VGSCs, the enzyme glutathione S-transferase (GST), ligand-gated chloride channels, and/or ATP-dependent transporters (e.g., multidrug-resistant protein) [30,40,48,49].

VGSCs are membrane proteins that play a crucial role in the generation and propagation of action potentials in neurons and other excitable cells. By binding to VGSCs, permethrin normally provokes a prolonged opening of these channels. This prevents the channels from closing rapidly, as they normally would after depolarization. The sustained opening of VGSCs results in a continuous influx of Na+ ions into the arthropod neurons, leading to persistent activation and subsequent neural hyperexcitation, thus resulting in the paralysis and eventual death of the scabies mites [30,40,48]. Mutations in the VGSC gene reduce permethrin binding activity and, consequently, the mite’s sensitivity to the neurotoxic effects of permethrin [40,48].

GSTs are a family of enzymes involved in the detoxification process of various endogenous and exogenous compounds, including insecticides like permethrin [40,49]. Heightened activity or expression of GST can be a result of genetic mutations or adaptive upregulation in response to prolonged exposure to both permethrin and ivermectin in different mite species.

Ligand-gated chloride channels are integral to the neurophysiology of *Sarcoptes scabiei*. These proteins mediate rapid inhibitory neurotransmission upon ligand binding, leading to chloride influx and hyperpolarization. Ivermectin exerts its acaricidal effect by potentiating glutamate- and GABA-gated chloride channels. This prolongs the channel open state, resulting in a continuous chloride influx and subsequent paralysis and death of the mite. The emergence of ivermectin resistance in *S. scabiei* may arise from alterations in the structure or function of these channels, or from downstream signaling perturbations [40,50].

Finally, increased expression of Multidrug Resistance-Associated Proteins (MDRP), which are molecules associated with resistance to several drugs, including chemotherapeutic agents, may also be linked to ivermectin resistance [49].

### 3.3. Alternatives Strategies in the Context of Drug Resistance

Considering the potential for resistance, there is a growing number of publications advocating multifaceted strategies to combat scabies. Studies have highlighted the need for better diagnostic capabilities [22,23,51,52], enhanced patient education, contact tracing, and public health interventions to prevent the spread of scabies and hence reduce the risk of resistance [22,33,52,53,54].

Therapeutic options reported in the literature encompass a broad spectrum. These include traditional treatments such as ivermectin [22,45,46,55,56], as well as emerging strategies such as higher doses of ivermectin [57], slow-release ivermectin formulations with potential therapeutic effect for up to six months [58], or topical ivermectin [20,37]. Combination therapies (e.g., permethrin with oral ivermectin [59] or benzoate benzyle with oral ivermectin [60,61]) have also been explored. Additionally, there is growing interest in reintroducing ancient therapies [46,62,63,64], utilizing natural products [65,66,67,68,69], and developing novel therapeutic approaches [16,62,63,65,66,70,71,72,73,74,75,76].

## 4. Discussion

The existence of true resistance to anti-scabies drugs is still debated by some authors, who suggest that patients’ failure to respond to treatment is often due to inadequate treatment, termed pseudo-resistance [77,78]. Clinicians should suspect drug-resistant scabies in patients with persistent or recurrent infestations despite appropriate treatment, particularly after multiple courses of first-line therapies like permethrin. Key indicators include ongoing pruritus, persistent lesions, or worsening symptoms two weeks post-treatment. Resistance may also be suspected if multiple cases within the same household or institutional setting fail to respond to treatment [43]. Risk factors such as limited patient mobility, prior topical steroid use, or immunosuppression may increase the likelihood of drug resistance [41]. Additionally, clinicians should rule out poor adherence to treatment protocols or reinfestation, both of which can mimic resistance [43]. A study involving 21 patients utilizing a fluorescent cream demonstrated that despite instructional guidance, none of the participants were able to apply the treatment correctly [79].

In this context, public and professional education on the proper use of scabicides is crucial to prevent resistance. Factors contributing to treatment failure include incorrect diagnosis, unclear information regarding treatment modalities, insufficient quantity of drugs and/or insufficient drug dosage, inadequate treatment duration, single application of permethrin or one-time ivermectin intake, suboptimal patient adherence and compliance, insufficient laundering of clothes and/or bedsheets at 60 °C, inadequate disinfection of affected furniture, failure to treat household members or close contacts, untreated body areas (such as subungual folds, which are frequently left untreated, highlighting the need to trim the fingernails very short [80]), and irritant contact dermatitis, particularly in the genital region, which may lead to treatment discontinuation [22,61].

Finally, it is important to note that many patients experience persistent pruritus following scabies treatment, often resulting in premature return visits. This phenomenon is likely due to the natural course of the disease, as pruritus and associated skin lesions may persist for 4-6 weeks post-treatment, even in the absence of active infestation.

While treatment failures can occur due to inadequate therapy, the emergence of scabies resistance is undeniable. A growing body of clinical, in vitro, and case report data supports the increasing prevalence of permethrin resistance [28,29,30,31,32,33,34,35,36,37,38,39]. Geographical disparities in permethrin resistance among scabies patients have been observed, likely influenced by factors such as local prescribing practices, treatment accessibility, and population dynamics. Regions with extensive permethrin use, such as certain parts of Australia, have reported higher resistance rates, highlighting the importance of ongoing surveillance to detect emerging resistance patterns, especially in areas with high scabies prevalence [28,29].

Given these data, it is crucial to reconsider permethrin as the primary treatment option in clinical practice guidelines. To effectively manage drug-resistant scabies, a multifaceted approach sounds essential. This includes enhancing diagnostic capabilities and implementing comprehensive public health strategies that address socio-economic factors linked to scabies outbreaks. Prioritizing infection control measures in high-risk settings such as nursing homes, prisons, and impoverished communities is also essential [22,33,52,53,54]. The emergence of resistance also underscores the urgent need for novel therapeutic strategies, including higher drug dosages, re-consideration of ancient therapies, combination regimens, or newly developed therapies (Table 1) [54,55,59,60,61,62,63,64,67,68,69,70,71,75,76].

Ivermectin (200 μg/kg of body weight as two doses one or two weeks apart) remains a valuable alternative to permethrin for scabies management. Most recent studies continue to support ivermectin’s high efficacy [45,46]. It has demonstrated effectiveness in mass treatment programs for both endemic and epidemic scabies, often providing a more cost-effective and practical approach compared to topical therapies [1,85].

Oral ivermectin may cause adverse effects, including headache, nausea, vomiting, dizziness, asthenia, paresthesia, hypotension, fever, chills, anorexia, rash, pruritus, edema, dyspnea, abdominal pain, gastrointestinal upset, myalgia, and arthralgia. Nevertheless, most ivermectin-related side effects observed in patients treated for scabies are mild and transient [1,45,46,85].

Recent studies suggest that ivermectin might be suitable for children aged 2–4 years weighing 10–14 kg, although specific dosing recommendations should be followed [58,84]. While observational studies have not identified apparent adverse pregnancy outcomes following inadvertent ivermectin exposure [5], the drug remains largely contraindicated during pregnancy due to insufficient human safety data [86]. This recommendation is primarily based on animal studies demonstrating adverse pregnancy outcomes (oral clefts and clubbed forepaws) in mice at doses significantly exceeding therapeutic human doses (150 μg/kg) [86].

Other considerable advantages of oral ivermectin include its ease of administration, improved patient adherence, and reduced risk of application errors compared to topical treatments, which are often time-consuming, inconvenient, and require multiple applications over several days [46].

The emergence of ivermectin-resistant scabies mites remains a potential threat [29,42]. However, unlike the relatively straightforward resistance landscape of permethrin, ivermectin resistance in scabies appears more complex. Ivermectin resistance in scabies contrasts with the more pronounced resistance observed in intestinal helminths of livestock, where widespread resistance has been documented [5]. While there are indications of increased ivermectin tolerance in scabies mites in laboratory settings [29,42,45,46], definitive clinical evidence of widespread ivermectin resistance in human scabies infestations is lacking. The clinical reports of ivermectin resistance among patients with scabies remain largely anecdotal, have been mainly reported in patients with crusted scabies, and are not necessarily recent [29,44].

To address the challenge of potential ivermectin resistance, a multifaceted approach is essential. This includes ongoing surveillance, judicious ivermectin use, and the exploration of alternative treatment strategies. Given the empirical basis for current ivermectin dosing in scabies and the successful use of increased doses in other parasitic infections like pediculosis, malaria, onchocercosis, and trichuriasis [57], exploring the efficacy of higher ivermectin doses for resistant scabies warrants consideration. To obviate the need for a second dose of ivermectin treatment, slow-release ivermectin formulations with potential therapeutic effect for up to six months [58] also represent a promising avenue for development. Topical ivermectin has been reported effective in a limited number of permethrin-resistant scabies cases [20,37]. In this perspective, the potential impact of increasing topical ivermectin use for other conditions, such as rosacea, on the development of scabies mite resistance warrants further investigation. Combination of ivermectin and permethrin [59] or ivermectin and benzyl benzoate [60,61] is another reported therapeutic option, which might offer synergistic effects, improve treatment outcomes, and delay the onset of resistance. However, further research is needed to demonstrate that this combination is both safe and more effective (i.e., not associated with an increased risk of drug resistance).

Benzyl benzoate is an ester of benzyl alcohol and benzoic acid, and has been used for decades for its acaricidal properties in the treatment of scabies [1,3,18]. Despite being overshadowed by newer agents like permethrin or ivermectin, benzyl benzoate remains in use, particularly in resource-limited settings, where it is often more accessible and cost-effective [1,3,18]. Although a German case report reported treatment failure with benzyl benzoate (as well as permethrin and ivermectin), characterized by persistent pruritus and infestation despite adherence to recommended regimens [47], there is currently no substantial evidence that scabies mites have developed resistance to benzyl benzoate. This is likely due to its distinct mechanism of action, which differs from that of other commonly used agents like permethrin, potentially reducing the likelihood of cross-resistance. While the precise mechanism is not fully understood, benzyl benzoate is believed to penetrate the exoskeleton of the mite and interfere with its nervous system [87].

Recent studies have indicated that benzyl benzoate may retain efficacy in cases of scabies that are resistant to first-line antiparasitic medications. A 2024 Austrian double-blind randomized control trial of topical permethrin vs. benzyl benzoate showed a significant superiority of benzyl benzoate 25% emulsion compared with permethrin 5% cream (87% vs. 27%), highlighting the importance of considering benzyl benzoate in the management of drug-resistant scabies and suggesting that benzyl benzoate is an appropriate first-line therapy in the treatment of scabies [62]. Another study from the same group showed that topical benzyl benzoate and oral ivermectin showed comparable good therapeutic efficacy [46].

While not available in the United States or Canada, benzyl benzoate is widely available outside North America. Benzyl benzoate has been shown to have a favorable safety profile, with the main adverse effect being local irritation, which is generally mild and self-limiting [46]. The application regimen, however, requires careful adherence to ensure efficacy, as improper use may lead to treatment failure. Recent data suggest that benzyl benzoate may be appropriate for patients younger than 2 years [81]. The safety data for benzyl benzoate during pregnancy remain limited [86,88].

Further research is needed to delineate the optimal use of benzyl benzoate, particularly in comparison with other therapeutic options in cases of drug-resistant scabies.

Sulfur ointment (2–10%) has re-emerged as a reasonable option to permethrin in the management of scabies, particularly in regions with high permethrin resistance or treatment failure rates. Upon topical application, sulfur is metabolized into hydrogen sulfide, polythionic acid, and pentathion, which possess antibacterial properties and scabicidal activity. Historically employed in Africa and South America, sulfur ointment is considered safe for pregnant women and infants by some authors [63,83]. Its efficacy, coupled with a favorable safety profile, has led to its inclusion in treatment guidelines for certain regions [16]. Recent studies suggest that sulfur ointment may even surpass permethrin in treatment efficacy [63]. Despite its cost-effectiveness and low risk of resistance development, sulfur ointment’s unpleasant odor, messiness, and potential for skin irritation may limit its acceptability.

Topical 10% crotamiton and 0.5% malathion are less effective than other treatments, and well-designed studies are limited [1]. Lindane may be associated with serious adverse events, including death and seizure, and is also classified by the International Agency for Research on Cancer as carcinogenic to humans [1,89]. Additionally, resistance to lindane and crotamiton has been reported [50]. Hence, it is reasonable to avoid these drugs.

Spinosad 0.9%, a neurotoxin previously used to treat head lice, was recently approved by the US Food and Drug Administration for the topical treatment of scabies [70]. This drug seems active after a single dose and shows significant efficacy with little systemic absorption and without serious adverse reactions, making it a promising treatment for classical scabies.

Moxidectin, a macrocyclic lactone closely related to ivermectin, is FDA-approved for onchocerciasis and shows promising results as a scabicide. Its extended half-life of 23 days potentially covers the entire life cycle of *Sarcoptes scabiei* and its superior skin penetration compared to ivermectin is advantageous. Preclinical studies in porcine models have demonstrated superior efficacy of moxidectin over a single dose of ivermectin (0.3 mg/kg) [75]. A clinical trial (NCT03905265) is currently underway to establish optimal dosing and efficacy for oral moxidectin treatment of scabies [76].

*Tinospora cordifolia*, a medicinal plant used for the treatment of tropical wounds and ulcers, has been shown to have clinical efficacy against scabies in one single-blinded, randomized trial [65]. Balsam of Peru is included among anti-scabies agents in a national guideline from Turkey [16] but clinical studies are lacking. Moreover, it often induces contact dermatitis. A variety of other agents, including tea tree oil, a known antimicrobial [67], eugenols [68,72], or other essential oils [69] have demonstrated in vitro scabicidal activity, likely by targeting the arthropods’ nervous systems. The lipophilic nature of essential oils is thought to play an important role in penetrating through the cuticle of arthropods, but randomized controlled clinical trials are lacking. *Ligularia virgaurea*, a genus highly diversified in the eastern Qinghai-Tibet Plateau region and adjacent areas, displays a strong acaricidal activity, but further studies are warranted to identify the active compounds in *Ligularia virgaurea* and to evaluate them in clinical trials, animal acute toxicity tests, and safety tests [66].

Keratolytic agents or acitretin might also be beneficial in treating crusted scabies [90,91,92].

Research into synergists, molecules that enhance permethrin’s efficacy, offers promising avenues to overcome scabies resistance [71,73]. These compounds target and inhibit key resistance mechanisms in mites, such as GSTs and cytochrome P450 monooxygenases. By co-administering these enzyme inhibitors with permethrin, mites’ ability to metabolize and detoxify the drug is significantly reduced, restoring permethrin’s effectiveness. Additionally, synergists can disrupt other resistance pathways, such as enhancing permethrin penetration or inhibiting drug efflux. These approaches have shown significant promise in clinical studies [73,74]. While clinical evidence is still emerging, these findings suggest that synergists could reduce necessary permethrin doses and the development of resistance. This strategy may revitalize permethrin as a first-line treatment, allowing ivermectin to be used as a last treatment option in order to avoid the development of ivermectin resistance.

Several vaccine candidates targeting various mite proteins and components, such as allergen and cuticle proteins, have been explored in preclinical studies. While these investigations demonstrated promising immunogenicity and protective efficacy in animal models, translation to human trials remains limited [93]. A significant challenge is that the introduction of these new therapeutic strategies may not be readily available or affordable in many endemic regions.

Limitations to this review include the heterogeneity of included studies, the lack of assessment of the risk of bias, the absence of standardized tool or checklist to assess the quality of individual studies, the paucity of details in some publications, and the potential for publication bias due to our focus on English-language peer-reviewed studies [94].

## 5. Conclusions

Scabies continues to be a substantial global public health concern, exacerbated by the growing prevalence of drug resistance, especially to permethrin. Combating this challenge requires a multifaceted approach encompassing public health interventions, revised therapeutic strategies, and updated guidelines. Ivermectin is an effective alternative to permethrin for scabies management, although the risk of resistance to this drug needs to be monitored. Benzyl benzoate and sulfur ointment are valuable options, particularly in resource-constrained settings. Spinosad is another promising alternative. Exploring synergistic drug combinations and developing effective vaccines represent long-term strategies for addressing this persistent issue.

## Figures and Tables

**Table 1:** Therapeutic options in case of suspected permethrin-resistant scabies for which human clinical studies are available.

Drug	General Posology Regimen	Side Effects	Pediatrics	Options
10 (children)–25% (adults) benzyl benzoate cream or lotion	Apply for 3 consecutive days [46,62]	Skin irritation	Probably appropriate for children < 2 years [81]	Combination with oral ivermectin [60,61]
2–12.5% sulfur cream or ointment	Apply overnight for 3–7 days [63,82,83] before bedtime from the neck to the soles of the feet and wash off the following morning. Repeat for 5 days	Bad odor, xerotic eczema	Safe at 6% concentrations in infants [82]	
Oral ivermectin	200 μg/kg of body weight as two doses one or two weeks apart	Mild and transient headache, nausea, vomiting, dizziness, asthenia, paresthesia, hypotension, fever, chills, anorexia, rash, pruritus, dyspnea, abdominal pain, gastrointestinal upset, myalgia, and arthralgia	Suitable in those > 15 kg and probably suitable for children aged 2–4 years weighing 10–14 kg [58,84]	Combination of ivermectin and permethrin [59] or ivermectin and benzyl benzoate [60,61], Topical 1% ivermectin for 7 to 14 h [20,37]
Spinosad 0.9% topical suspension	Single application and leave on the skin for at least 6 h before washing	Skin irritation, dry skin	Approved for children aged 4 years	

## Data Availability

The authors confirm that they have full access to all the data in the study and take responsibility for the integrity of the data and the accuracy of the data analysis.
